# Evaluation of Two Formulated Chitin Synthesis Inhibitors, Hexaflumuron and Lufenuron Against the Raisin Moth, *Ephestia figulilella*

**DOI:** 10.1673/031.012.10201

**Published:** 2012-08-25

**Authors:** Simin Khajepour, Hamzeh Izadi, Mohammad Javad Asari

**Affiliations:** ^1^ Department of Plant Protection, Vali-e-Asr University of Rafsanjan, Iran; ^2^ Azad Islamic University, Jahrom, Iran; ^3^ Plant Pests and Diseases Research Center, Kerman, Iran

## Abstract

The raisin moth, *Ephestia figulilella* Gregson (Lepidoptera: Pyralidae), has a nearly cosmopolitan distribution, and causes severe quantitative and qualitative losses throughout the world. The larvae attack various drying and dried fruits, fallen figs, and damaged or moldy clusters of grapes on vines. Control of this pest in storage depends mostly on synthetic pesticides with several adverse side effects. To mitigate the adverse effects of these pesticides, investigations have focused on the development of compounds with more selectivity, and short residual life. In this research, insecticidal effects of two chitin synthesis inhibitors, hexaflumuron and lufenuron, were investigated against *E. figulilella*. Graded concentrations of each pesticide were prepared with distilled water. One-day-old fifth instar were sprayed by Potter's precision spray tower. Application of hexaflumuron and lufenuron on last instar larvae of *E. figulilella* caused not only mortality in larval stage, but also caused defects in pupal and adult stages. Larval mortality increased as concentration increased. The longevity of the fifth instars in both hexaflumuron and lufenuron treatments, in comparison with the controls, increased by more than 12 days. The longevity of adults decreased by about 10 days. Probit analysis data revealed that the sensitivity of the test insect to hexaflumuron (EC_50_ = 95.38 ppm) was greater than lufenuron (EC_50_= 379.21 ppm).

## Introduction

The most economically-important stored products in the southeast of Iran are pistachio and date. They may be stored for periods of a few weeks to a few years before they are fed or processed. These dried foods may become infested with several stored-product pest insects. Among these pests, the raisin moth, *Ephestia figulilella* Gregson (Lepidoptera: Pyralidae), is a primary and destructive pest, and causes severe quantitative and qualitative losses throughout the world. The larvae feed on dried fruits of all types, and also on freshly harvested carobs and meals. Alleviation of this pest problem in storage relies mostly on synthetic pesticides. Most of the insecticides currently used for the control of insect grain pests are neurotoxic compounds, primarily methyl bromide and phosphine, which have several adverse side effects including toxicity to nontargets or grains, development of pest resistance, and resurgence and environmental contamination (Talukder and Howse 1993; Subramanyam and Hagstrom 1995). Control of raisin moth depends mostly on fumigation with methyl bromide or phosphine. Some organophosphorus insecticides (i.e., chlorpyrifos, diazinon, dicapthon, fenitrothion, and fenthion) have been evaluated against this pest (Strong 1969; Buchanan et al. 1984). In the last two decades, investigations have been focused on the development of compounds that have more selectivity and a short residual life. Consequently, a number of insecticides with novel modes of action have been developed. These include a new class of chitin synthesis inhibitors, juvenile hormone (JH) analogous, ecdysone agonists, neo-nicotinoids, and botanical insecticides such as azadirachtin. In view of the above, it is essential to evaluate these new compounds in the laboratory in order to obtain data on their efficacy, as well as their possible use in integrated pest management. Since these molecules have different modes of action and are non-neurotoxic, it is essential to realize their effects on insect development, reproduction, and other physiological processes (Sun and Barrett 1999).

In recent years, the toxicity of insecticides to humans and wildlife has caused much public concern, and led to the use of more target-specific chemicals (Paoletti and Pimentel 2000). Because of the secondary effects of conventional insecticides, the insect growth regulators are receiving more practical attention, in hopes to provide safer foods and a cleaner environment. The benzoylphenyl ureas, hexaflumuron, and lufenuron are insect growth regulators that interfere with chitin synthesis, disrupt hormonal balance with exchanging in molting process, and inhibit the insect's growth (Oberlander and Silhacek 1998). Keeping this point in view, the present study is proposed to undertake detailed toxicological investigation on the effect of two chitin synthesis inhibitors, hexaflumuron and lufenuron, with the following objectives: (1) Detailed dose-effect-relationship study to obtain the lethal concentration (LC_50_) on the last instar of *E. figulilella*, and (2) to obtain the effective concentration (EC_50_ morphological) of *E. figulilella* at different concentrations.

## Materials and Methods

### Insect culture and larval handling

A colony of the test insect, *E. figulilella*, was established in the insectary from field collected, disease-free moths, and reared under constant temperature of 26 ± 2° C, 65 ± 5% RH, with a photoperiod of 16:8 L:D. The adults were fed on a solution of 20% honey, and larvae were fed on a semi-synthetic diet composed of date powder (400 g), wheat flour (400 g), honey (150 g), yeast (25 g), and glycerin (120 mL).

### Insecticide

Commercial formulations of hexaflumuron (Consult, 10%, Dow AgroSciences, http://www.dowagro.com/) and lufenuron (MATCH %50 EC, Syngenta Crop Protection, http://www.syngentacropprotection.com/) were used. Based on preliminary tests, graded concentrations of each pesticide (62.5, 125, 350, 375, and 500 ppm for hexaflumuron, and 500, 750, 1000, 1250, and 1500 ppm for lufenuron) were prepared with distilled water. Each treatment consisted of five concentrations and a control (distilled water).

### Bioassay

One-day-old fifth instar larvae were transferred into Petri dishes, and sprayed with 2 mL of aqueous emulsions of different concentrations of each insecticide. The spray was applied at 10 mbar using a Potter's precision spray tower (Burkard Manufacturing Co. Ltd., http://www.burkard.co.uk/). This experiment was repeated 3 times for each treatment with 10 larvae. Distilled water was used as control. Treated larvae were maintained in a climate chamber set at 26 ± 2° C, 65 ± 5% RH, with a photoperiod of 16:8 L:D, and reared on a semi-synthetic diet. Larval mortality, defective pupae, pupal mortality, defective adults, and larval-pupal intermediates were recorded. The percentage of larval mortality and normal adults emerged were taken as the criteria for studies on dose-effect relationship.

### Data analysis

The results were expressed as percent of larval mortality, abnormality, and adult emergence. Probit analysis was used for estimation of LC_50_ and EC_50_ by POLO-PC 2002 software.

The percentage of larval mortality was used as the criterion for estimation of LC_50_ value, and the percentage of normal adults emerged was used as the criterion for estimation of EC_50_ value.

## Results and Discussion

In this study, effects of two chitin synthesis inhibitors, hexaflumuron and lufenuron, were investigated on larval mortality, morphology, and longevity of the fifth instar larvae, pupae, and adults of *E. figulilella*. Application of hexaflumuron and lufenuron on last instar larvae of *E. figulilella* caused not only mortality in larval stage, but also caused defects in pupal and adult stages, and in some cases produced larval-pupal intermediates. Malformed pupae were not able to form a pupal case, and never emerged as adults. Some larvae molted into malformed supernumerary larvae. Treated larvae became dark in the posterior end of the abdomen. These larvae were not able to feed, and eventually died. These results are consistent with results of Hughes et al. (1986) and Karimzadeh et al. (2007). Molting and metamorphosis are two critical physiological events in the life of insects. All insects molt periodically in order to grow, and all but a very few go through either gradual or complete metamorphosis to become an adult. These two events are regulated by the steroid 20-hydroxyecdysone, and the sesquiterpenoid juvenile hormone (Nation 2008). It is obvious that any interference with the homeostasis of these two hormones with exogenous sources of the hormones or synthetic analogs can be exploited as a novel insecticide, targeted to disrupt normal development of pest insects (Aribi et al. 2006).

In both hexaflumuron and lufenuron treatments, larval mortality increased as concentration increased. In the other words, larval mortality was proportional with pesticide concentration (Table 1). Longevity of the last instar larvae treated with hexaflumuron and lufenuron (about 20 days) was significantly longer than the control larvae (8 days). Hexaflumuron and lufenuron as chitin synthesis inhibitors induce morphological disruptions at molt. In our study, treatment of early last instar larvae with these two pesticides resulted in lengthening the longevity of these larvae, and in the formation of malformed prepupae that had both larval and pupal characters. These findings are in agreement with results of Behroozi et al. (2011). Investigations of Omatsu et al. (1991) showed that five days after treatment of the common cutworm, *Spodoptera litura*, larvae with chlorfluazuron, various degrees of malformations in the integuments at/after molting were observed. Significant abnormalities were also found in adults of Phlebotomine sand flies treated with chlorfluazuron as third instar larvae (Quesada and Montoya-Lerma 1994). These results are consistent with our findings. In our study, treatment of fifth instar larvae of *E. figulilella* with hexaflumuron and lufenuron significantly decreased longevity of adult moths compared to the control (2–4 days for treatments and 11–15 days for control). Saenz-de-Cabezon et al. (2006) evaluated the effect of the chitin synthesis inhibitor lufenuron against different developmental stages of *Lobesia botrana*, and found that when lufenuron was fed to adults at 10 ppm, it reduced their fecundity and fertility, but it did not affect adult longevity.

Insecticide bioassay refers to any quantitative procedure used to determine the relationship between the amount (i.e., dose or concentration) of an insecticide administered and the magnitude of response in a living organism. Insecticide bioassay with insects or other arthropods is often used to estimate the median lethal concentration (LC_50_). The LC_50_ is the concentration of an insecticide required to kill 50% of a given population or strain under the specified conditions. In some instances, the toxicity of an insecticide is measured in sublethal effects (i.e., effects on growth, reproduction, etc.) instead of mortality. In such cases, the term median effective concentration (EC_50_) may be used to express the toxicity level of the insecticide (Zhu 2008). Application of insect growth regulators at certain periods during the life of insects will adversely affect metamorphosis and other hormonal functions, causing mortality or producing abnormal insects that fail to reproduce. So, the concentration that caused 50% reduction in normal adult emergence was considered as EC_50_.

**Table 1.**
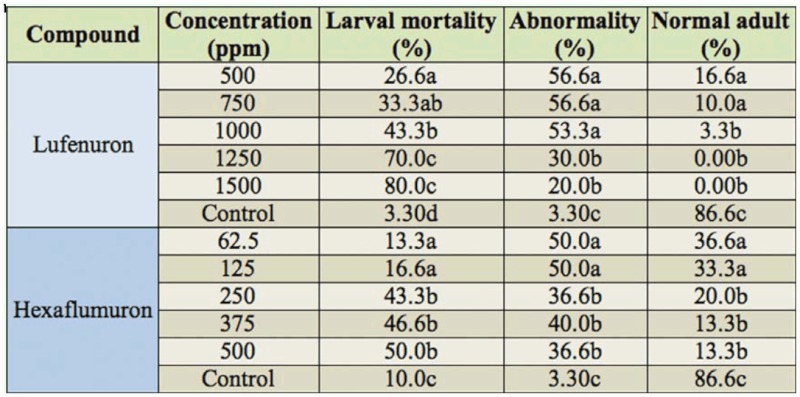
Larval mortality, abnormality, and normal adult emergence (%) of *Ephestia figulilella* when fifth instar larvae were treated with lufenuron and hexaflumuron

**Table 2.**
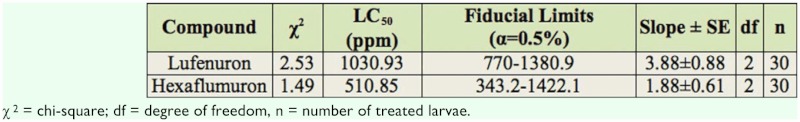
Evaluation of hexaflumuron and lufenuron against final instar larvae of *Ephestia figulilella* (larval mortality was used as a criterion).

**Table 3.**
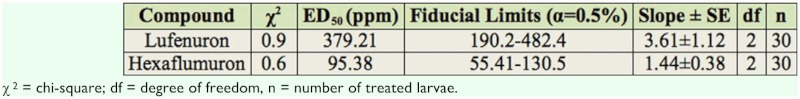
Evaluation of hexaflumuron and lufenuron against final instar larvae of *Ephestia figulilella* (adult emergence was used as a criterion).

When larval mortality was taken as a criterion for comparison of hexaflumuron and lufenuron, probit analysis data revealed that the sensitivity of the test insect to hexaflumuron (LC_50_ = 510.85 ppm) was more than lufenuron (LC_50_ = 1030.93 ppm) (Table 2). In another set of experiments, larval-pupal mortality, and defective prepupae, pupae, and adults, were considered (only normal adult emergence was taken as criterion for comparison), and the EC_50_ was used as standard (Table 3). Hexaflumuron with the EC_50_ value of 95.38 ppm was found to be more effective against *E. figulilella* than lufenuron with EC_50_ value of 379.21 ppm. It may be concluded from these results that *E. figulilella* is more sensitive to hexaflumuron than lufenuron. Karimzadeh et al. (2007) rated the toxicity of five different chitin synthesis inhibitors in the following order: hexaflumuron > lufenuron > diflubenzuron > triflumuron > cyromazine. Saenz-de-Cabezon et al. (2006) found significant effects of lufenuron against different larval instars of *L. botrana*.

Rate of change of effect in relation to a unit change in concentration is expressed by the slope of a line, which in turn expresses the variability in susceptibility of the test population. A steep line means a population has a small variation in susceptibility, whereas a flat line means a population varies widely in susceptibility. The slope of the dose-response line of the lufenuron was steep, and the difference between the highest and lowest concentrations was low. That means the population was homogeneous in susceptibility, and with a fairly small increase in insecticide concentration, the mortality would increase considerably. This finding necessitates more careful use of these chitin synthesis inhibitors in the field in order to prevent exerting a high selection pressure that could eliminate the susceptible individuals, and lead to selection of resistant individuals. The slope of the dose-response line of the hexaflumuron was flat, and the difference between the highest and lowest concentrations was high. That means with a fairly large increase in insecticide concentration, the mortality would increase considerably (Robertson et al. 2007).

Hexaflumuron and lufenuron both belong to the same group of insect growth regulators, and have the same mode of action, but their effects on the same species are quite different. In most of the studies that have been conducted on the effects of these two pesticides, hexaflumuron has been found to be more effective against the same pest than lufenuron. For example, results of Karimzadeh et al. (2007) indicated that among the chitin synthesis inhibitors tested, hexaflumuron and lufenuron (with LC_50_ values of 0.79 and 27.3 mg ai/L respectively) were the most effective at low concentrations against the *Leptinotarsa decemlineata*. These results show that *L. decemlineata* is 34.5 times more sensitive to hexaflumuron than it is to lufenuron. Su and Scheffrahn (1996) reported that the overall potential of lufenuron as a bait toxicant is less than that of hexaflumuron.

## Abbreviations

EC50,: median effective concentration;
LC50,: median lethal concentration
